# Glycin-rich antimicrobial peptide YD1 from *B. amyloliquefaciens,* induced morphological alteration in and showed affinity for plasmid DNA of *E. coli*

**DOI:** 10.1186/s13568-016-0315-8

**Published:** 2017-01-03

**Authors:** Md. Saifur Rahman, Yun Hee Choi, Yoon Seok Choi, Jin Cheol Yoo

**Affiliations:** Department of Pharmacy, College of Pharmacy, Chosun University, Gwangju, 501-759 Republic of Korea

**Keywords:** Antimicrobial peptide, *Bacillus amyloliquefaciens CBSYD1*, Purification, Mechanism of action

## Abstract

Antimicrobial peptides (AMPs), low-molecular-weight proteins with broad-spectrum antimicrobial activity, are the most promising candidates for the development of novel antimicrobials. A powerful cationic glycine-rich AMP YD1 (MW ~ 1.0 kDa) was purified from *Bacillus amyloliquefaciens CBSYD1* isolated from traditional Korean fermented food kimchi, for the treatment of multidrug-resistant (MDR) bacteria. Strain *CBSYD1* was identified 99.79% similar to *Bacillus amyloliquefaciens* subsp. *plantarum FZB42(T)* by 16S rRNA sequence analysis. The amino acid sequence residues of YD1 were determined to be Ala-Pro-Lys-Gly-Val-Gln-Gly-Pro-Asn-Gly by Edman degradation method. After the analysis and comparison of YD1 peptide sequence using several bioinformatic servers, peptide sequence has been considered to be unique. YD1 displayed antimicrobial activity against gram-positive and gram-negative bacteria. The minimal inhibitory concentrations (MIC) of YD1 for *Escherichia coli KCTC1923 (E. coli), methicillin*-*resistant Staphylococcus aureus B15 (MRSA)*, and *vancomycin*-*resistant enterococci (VRE)* ranged from 8 to 64 µg/mL, representing greater potency than commercial reference antibiotics. The antimicrobial mechanism of YD1 was determined to involve cell-penetrating translocation inside the cell and interaction with the DNA leading ultimately to bacterial cell death. Analogously, Gly-Pro-Asn-Gly is the likely expected cell-penetrating motif for YD1. YD1 could be a promising antimicrobial agent for the clinical application.

**Electronic supplementary material:**

The online version of this article (doi:10.1186/s13568-016-0315-8) contains supplementary material, which is available to authorized users.

## Introduction

Natural products are extensively used in our society. More than 60% of approved drugs and drug candidates either are natural goods and/or derived from them. About 100,000 secondary metabolites (organic compounds, not directly involved in the growth, development, or reproduction of an organism) with molecular weight less than 2500 Da have been characterized; half from microbes and the other half from plants (Strobel and Daisy 2003). AMPs and “bacteriocins” have gained attention for their potential application in controlling pathogenic bacteria and infectious diseases. AMPs are ubiquitously produced by a wide range of microorganisms. *Bacillus*, a genus of significant interest to human health, produces a varied array of AMPs with several different basic chemical structures. Several peptides with biological activities have been identified from *Bacillus* and are under active investigation for their antimicrobial effects (Dischinger et al. 2009; Teixeira et al. 2009; Wu et al. 2005). Bacteriocin or bacteriocin-like substances have also been described for some other important pathogens such as *Listeria monocytogenes* and *Streptococcus pyogenes* (Cherif et al. 2001).

The history of use of fermented vegetables as a source of beneficial bacteria is extensive. For over 2000 years, Koreans have consumed salted and fermented vegetables such as kimchi. The primary ingredients of kimchi are *Baechu* cabbage and radish; however, other vegetables such as green onion, leek, and cucumber are also used for preparing various types of kimchi. Kimchi contains high levels of vitamins, minerals, dietary fibers, and other functional ingredients. Many previous studies have reported that kimchi has anticancer, antimicrobial, antioxidant, antiatherosclerotic, antidiabetic, and antiobesity effects among others (Islam and Choi 2009; Kim et al. 2007, 2011).

The rapid development and spread of bacterial resistance and the emergence of multi-drug resistant pathogens have created a need for the discovery of new classes of antimicrobial agents against novel microbial targets while evading existing resistance mechanisms. Elucidation of the mechanism(s) of action of antimicrobial drugs helps to characterize the interaction of the pathogen with both the chemical and the host, design improved antimicrobials, determine effective combinations of drugs, and understand the development of microbial resistance. The exploration of action mechanisms of developmental compounds, not originated from target-based discovery, should be undertaken early in their development stage to facilitate the modification of the drug scaffold for improved selectivity of action and pharmaceutical profile. The production of AMPs from bacteria isolated from fermented foods and their mechanisms of action have not been extensively studied.

In the present work, we designed a two-step study. Firstly, we isolated the strain from kimchi, identified, characterized, and purified the AMP, and further characterized the AMP designated as YD1. Secondly, upon confirmation of the novelty of purified YD1, we investigated its antimicrobial mechanism.

## Materials and methods

### Materials

DEAE-Sepharose Fast Flow and Sephadex G-25 columns were obtained from Pharmacia (Uppsala, Sweden). Bacterial media de Man-Rogosa-Sharpe (MRS) and Mueller-Hinton (MH) were purchased from Becton–Dickinson, Spark, USA. Agar was purchased from Daejun Chemicals and Metals Co, Gyeonggi-do, South Korea. All other reagents were of the extra pure grade. Strain *CBSYD1* was isolated from fermented kimchi.

### Culture media for YD1 production

The impact of various nutrient sources (carbon, nitrogen, and metal ion) on the antimicrobial compound production was determined and media optimization was performed according to our previous report (Cho et al. 2012). Fermentation was carried out in 50 mL media in 250-mL Erlenmeyer flasks with constant shaking at 160 rpm. Zone of inhibition was observed against *Mycobacterium smegmatis ATCC 9341* at every step of media optimization. Commercially available MRS and MH broth media were used as control media.

### Bacterial strain isolation and identification

Cabbages, from different provinces of Korea, were collected and processed for biochemical and molecular identification of microorganisms. The strain identification, based on morphological characteristics, was made according to Bergey’s manual of systematic bacteriology (Lechevalier 1989). Furthermore, the identification was confirmed by 16S rRNA sequence analysis and phylogenetic tree.

The nucleotide sequence of strain CBSYD1 was submitted to the GenBank (ncbi.nlm.nih.gov/Genbank) under the accession no. KY062987.

### Antimicrobial activity

A filter paper disc (8 mm, Toyo Roshi Kaisha, Japan) saturated with antimicrobial sample (40 µL) was placed on the surface of petri dish (87 mm × 15 mm) containing Mueller Hinton Agar (MHA). The plate was incubated at 37 °C, and a clear zone of inhibition surrounding the paper disc was measured in millimeter (mm).

An arbitrary unit per milliliter (AU/mL) was defined as the reciprocal of the dilution after the last serial dilution that resulted in an inhibition zone. The titer of the antimicrobial substance solution, in AU/mL, was calculated as (1000/d) D, where D was the dilution factor, and d was the dose, the amount of antimicrobial substance solution added to each spot. AU and the zone of inhibition were measured against *Mycobacterium smegmatis ATCC 9341* while optimizing *CBSYD1* media.

The minimal inhibitory concentration (MIC) was determined according to the method described by Weigand et al. (2008).

### Purification of YD1

*CBSYD1* was cultured for 36 h in optimized media (1% peptone, 2% maltose, and 0.01% CaCl_2_). The cultured supernatant was mixed with ammonium sulfate (30–80% saturation) and kept at 4 °C with overnight stirring. The precipitate was collected by centrifugation at 10,000 rpm for 30 min and re-dissolved in 10 mM Tris–HCl buffer (pH 7). The dialyzed sample was applied to a DEAE-Sepharose Fast Flow column (2.5 × 14 cm) pre-equilibrated with 10 mM Tris–HCl buffer, pH 7. The column was washed with the same buffer and eluted with a linear gradient of KCl (0–1 M). Fractions of 3 mL were collected at a flow rate of 0.3 mL/min. Active fractions were pooled, concentrated, and further purified with Sephadex G-25 column (1.5 × 28 cm) using the same buffer system.

### Electrophoresis and in-situ analysis

The molecular weight of peptide was determined by tricine SDS-PAGE (Schägger 2006). The in situ analysis was performed against indicator organism (~5 × 10^5^ cfu/mL) by overlaying the processed gel from tricine SDS–PAGE [after washes with 50 mM Tris/HCl buffer (pH 7.5) containing 2.5% Triton X-100 for several times] on 0.6% agar on Mueller–Hinton (DIFCO, USA) media and incubated at 37 °C.

### Amino acid sequencing and computational analysis

Amino acid sequence of YD1 was determined by Edman degradation using a Procise Model 492 protein sequencer (Applied Biosystems, CA, USA). The amino acid sequence was analyzed using BLAST search against GenBank (http://www.ncbi.nlm.nih.gov/BLAST) and Antimicrobial Peptide Database (http://aps.unmc.edu/AP/main.php). The 3D structure projection was predicted by I-TASSER (http://zhanglab.ccmb.med.umich.edu/I-TASSER/) under the job ID S281576.

### Stability of YD1

The thermal stability of YD1 samples was determined by exposure to 20, 40, 60, 80 and 100 °C for 30 min and to 121 °C/105 kPa for 15 min before analyzing the residual activity. Similarly, pH stability was determined over a range of pH 2–10 using 1 M NaOH or HCl. The effect of protease enzyme on YD1 stability was determined at two different enzyme concentrations (1 and 2 mg/mL).

### Cytotoxicity

Cytotoxicity assay was performed according to our previous report by Choi et al. (2016). The murine macrophage cell lines Raw 264.7 were seeded in 96-well plates and 24 h later, treated with purified YD1, concentration ranging 8–120 µg/mL.

### Synergism or antagonism of YD1 with antibiotics

YD1 was investigated for the interaction with antibiotics such as erythromycin (a protein synthesis translocation inhibitor), ceftriaxone sodium (a cell wall synthesis inhibitor), and a quinolone (ciprofloxacin) that interferes with DNA gyrase supercoiling.

Log phase-grown *E. coli* and *MRSA* were cultured in MHB at 37 °C and diluted to a final inoculation density of 1 × 10^5^–1 × 10^6^ cfu/mL in a total of 200 µL. The inhibition pattern indicates the interaction between the two compounds and the method enables the calculation of a fractional inhibitory concentration index (FICI), a numerical interpretation of the type of interaction displayed.

For wells containing the lowest inhibitory combination of drugs, a fractional inhibitory concentration (FIC) is derived for each well from the following calculation:

$$\begin{aligned} &= \frac{\text{MIC of compound A with B}}{\text{The MIC of compound A alone}}+ \frac{\text{MIC of compound B with A}}{\text{The MIC of compound B alone}}\end{aligned}$$

The FIC Index of ≤0.5 was considered to indicate synergism, a value ≥4 to indicate antagonism and all values >0.05 to <4.0 indicated an indifferent interaction (Ji 1996; Pasquale and Tan 2005; Williams 2001).

### Time-kill interaction between an antibiotic and YD1

Time-kill assays were conducted with concentrations corresponding to the MIC values of YD1 and erythromycin for reference strain, *E. coli*. Concentrations ranging from 8 to 256 µg/mL of YD1 or erythromycin were added to a bacterial suspension (1 × 10^5^–1 × 10^6^ cfu/mL) of the tested bacterial strain. Then, 1 mL of the tested suspension sample was collected every 1 h for viable cell counting in MHA plate followed by incubation at 37 °C for 24 h.

### Lysis of gram-negative spheroplasts

Spheroplasts are gram-negative bacteria in which nearly all of the outer membrane (OM) has been removed. Lysozyme destroys the peptide bonds in peptidoglycan and weakens the cell wall. *E. coli* was grown in 10 mL of MHB, incubated overnight at 37 °C on a shaker at 180 rpm. Each culture (100 µL) was used to inoculate 20 mL of fresh media and incubated at 37 °C for 2 h at 180 rpm. Spheroplasts were prepared as described by Kikuchi et al. (2015).

Re-suspended spheroplasts and whole cells suspension were adjusted to OD 570 nm of 0.2 and 100 µL of each was added to a clear, flat-bottomed microtiter plate in duplicate wells. Ten microliters of YD1 in 10 mM Tris–HCl buffer (pH 7) was added to test wells for both preparations (spheroplasts and whole cells) at the indicated different final concentrations. To the control, 10 µL of 10 mM Tris–HCl buffer (pH 7) was added in lieu of YD1. The percentage of intact spheroplasts or whole cells was calculated as:

$$= \left( {\frac{\text{Sample OD at time X}}{{{\text{Sample OD at time }}0}} } \right) \times {{100}}$$

Decrease in the OD of the suspension after addition of a membrane-active agent indicates lysis of spheroplasts.

### DNA binding assay

The plasmid DNA (150 ng) of *E. coli* was incubated with increasing concentration of peptides in 20 µL of binding buffer [5% glycerol, 10 mM Tris–HCl (pH 7), 1 mM EDTA, 1 mM DTT, 20 mM KCl, and 50 µg/mL BSA]. The reaction mixtures were kept at room temperature for 30 min, followed by addition of 4 µL of native loading buffer. An aliquot of 12 µL was applied to a 1% agarose gel, and electrophoresis was performed in 0.5 X tris–borate-EDTA buffer.

### Transmission electron microscopy (TEM)

TEM analysis was performed according to the method described by Lee et al. (2013). Ten milliliters of 10^6^ cfu/mL *E. coli* suspension was exposed to 5× MIC (40 µg/mL) of YD1 to observe morphological changes and calculate the percentage killing of *E. coli* cells.

## Results

### Strain isolation and identification

**Fig. 1:**
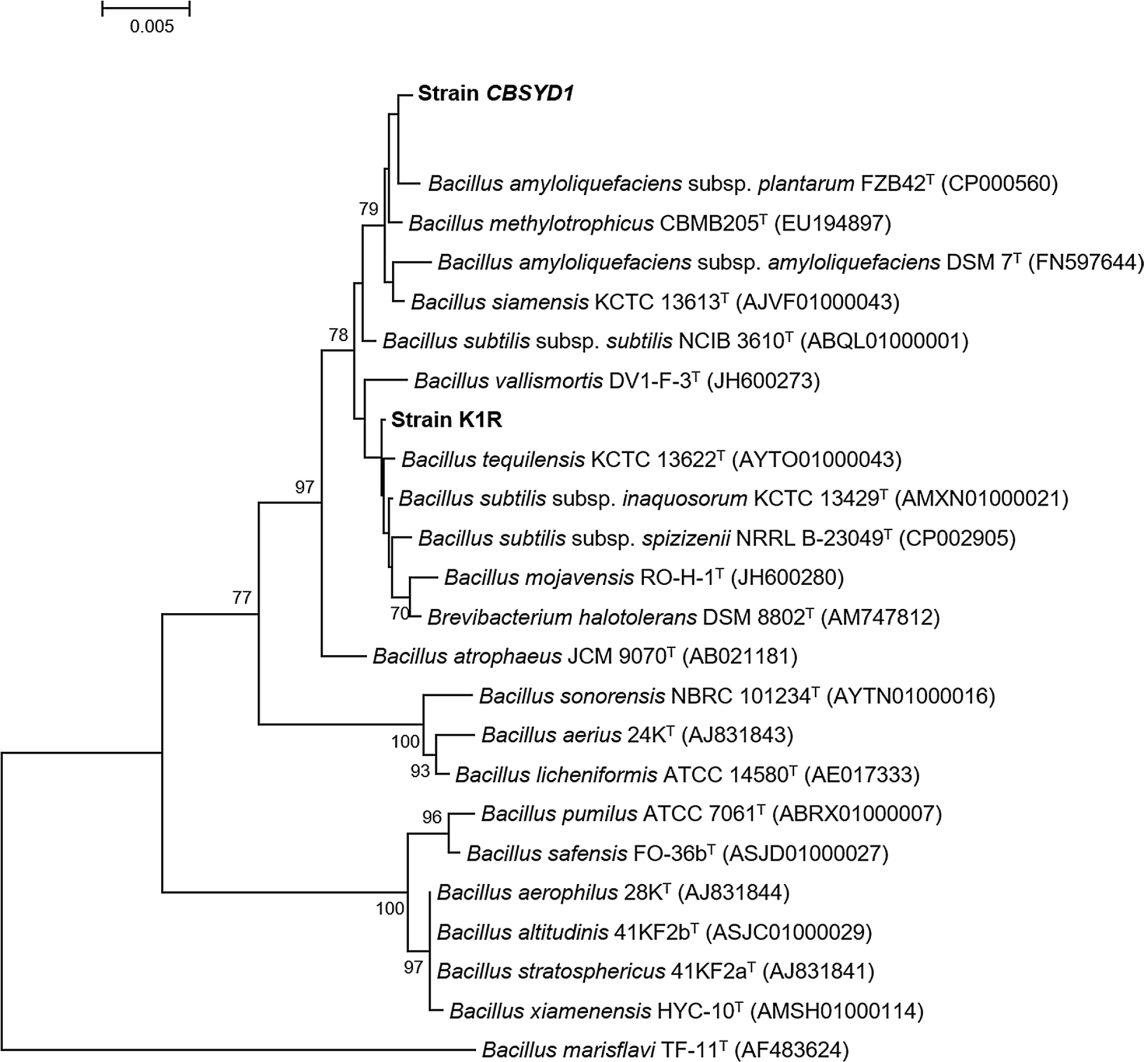
Neighbor-joining tree based on nearly complete 16S rRNA gene sequences showing relationships between *CBSYD1* and some closely related taxa of the genus *Bacillus*. The percentage numbers at the nodes are the levels of bootstrap support based on neighbor-joining analyses of 1000 resampled data sets

The strain showed a high degree of identity with many *Bacillus* strains in 16S rRNA gene analysis. The closest identity was with *Bacillus amyliquefaciens* subsp. *plantarum FZB42* (99.79%) (Accession no. CP000560). Because of a high level of gene similarity, along with identical morphological characteristics, the strain *CBSYD1* was identified and classified as *Bacillus amyliquefaciens CBSYD1*. A phylogenetic tree prepared from the 16S rRNA sequence has been presented in Fig. 1. Strain *Bacillus amyliquefaciens CBSYD1* has been deposited at the Korean collection for type culture (KCTC), which is belong to World Data Centre for Microorganisms (WDCM), under the accession number KCTC18507P.

### Culture media

The effect of various components of nutrition media on the production of the antimicrobial compounds in the crude sample were emphasized. Among carbon sources, 2% maltose promoted maximum production and among nitrogen sources, it was 1% peptone. Magnesium chloride or sodium chloride (in 36 h) seemed to be the best among minerals. Finally, media containing 1% peptone, 2% maltose, and 0.01% CaCl_2_ were determined to be optimum for the maximum antimicrobial production at 37 °C after 36 h over commercially available MRS and MH broth media (Additional file 1: Figure S1).

### Production and antimicrobial activity of YD1

**Fig. 2:**
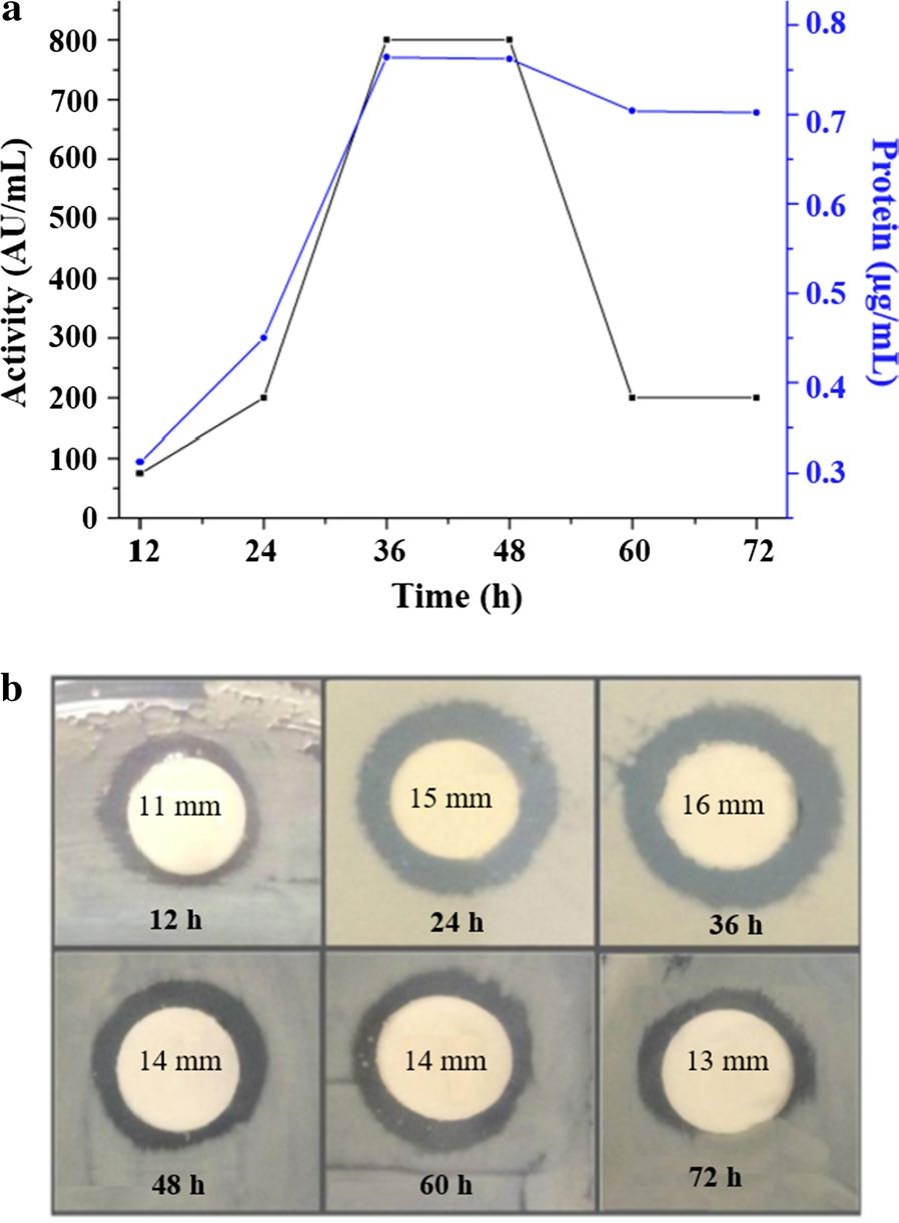
Production of bacteriocin and the inhibition activity against *Mycobacterium smegmatis ATCC 9341*. Total protein content (*black square*) production increased hourly and antibacterial activity AU/mL (*down arrow*) is maximum at 36 h (**a**). The zone of inhibition against *Mycobacterium smegmatis ATCC 9341* at every 12 h is shown (**b**). Cultivation was carried out in 250-mL flasks with 50 mL media, at pH 7 and 37 °C, with shaking at 160 rpm

The maximum production of YD1 was achieved in optimized media at 37 °C with shaking at 160 rpm for 36 h as shown in Fig. 2. The antibacterial activity (AU/mL) of YD1 lasted for 36 h (Fig. 2a) and up to 16 mm of a clear zone of inhibition was observed (Fig. 2b). Protein concentration increased considerably from 24 h (Fig. 2a) onwards. The antibacterial effects of YD1 against various gram-positive and gram-negative pathogenic bacteria were evaluated. YD1 was effective against *Staphylococcus aureus*, MRSA, and VRE with MIC values of 32, 16, and 32 µg/mL respectively; *E. coli* was found to be very sensitive (8 µg/mL) to YD1 whereas reference commercial antibiotics exhibited MIC values >128 µg/mL (Table 1).

**Table 1:** Antimicrobial spectrum of YD1

Microorganism	MIC (μg/mL)		
	YD1	Bacitracin	Vancomycin
*Gram*-*negative bacteria*			
* Alcaligenes faecalis ATCC 1004*	>128	>128	>128
* Salmonella Typhimurium KCTC 1925*	32	64	32
* Escherichia coli KCTC 1923*	8	>128	32
* Pseudomonas aeruginosa KCTC 1637*	16	>128	>128
*Gram*-*positive bacteria*			
*Enterococcus faecalis ATCC 29212*	64	2	0.5
*Bacillus subtilis ATCC 6633*	64	64	2
*Staphylococcus aureus KCTC 1928*	32	>128	>128
*Micrococcus luteus ATCC 9341*	64	64	1
*Mycobacterium smegmatis ATCC 9341*	8	>128	1
*MRSA B15*	32	64	>128
*VRE 2*	64	32	>128
*VRE 5*	64	64	>128
*VRSA*	>128	>128	>128

### Purification, molecular weight, and stability of YD1

**Fig. 3:**
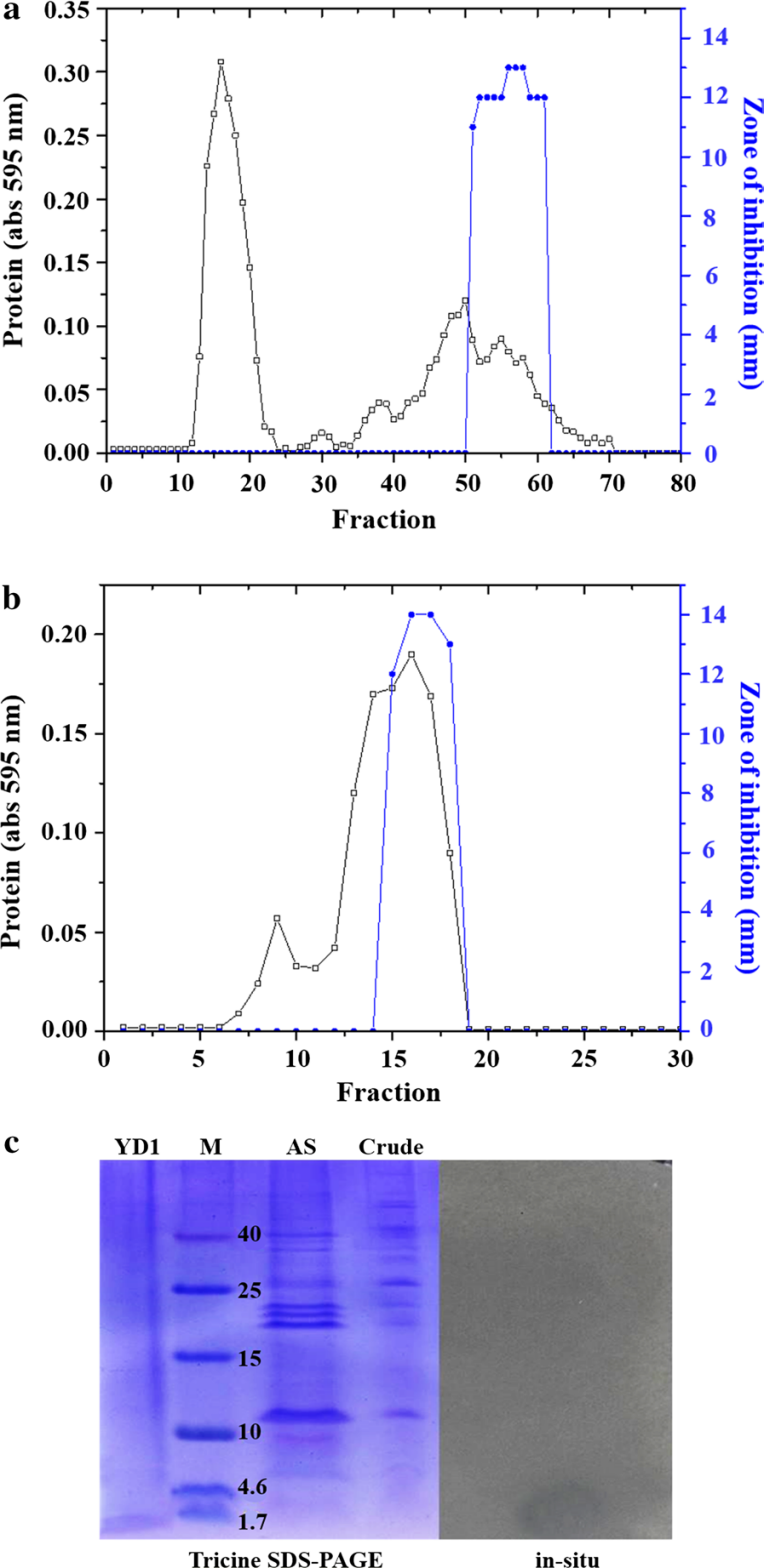
The elution profile of YD1 from a DEAE Sepharose Fast Flow ion exchange column (2.5 × 14 cm) (**a**) and a Sephadex G-25 permeation column (1.5 x 28 cm) (**b**). Tricine SDS-PAGE and in situ analysis. *Lane 1*, Sephadex G-25 pooled purified YD1; *lane 2*, protein marker (M); *lane 3*, 30–80% ammonium sulfate (AS) precipitation fraction; and *lane 4*, crude sample. Inhibition zone observed in the in situ assay is shown in the *right panel* (**c**)

Production of YD1 was carried out in optimized media. The purification of YD1 from the 36 h cultured supernatant (ammonium sulfate; 30–80% saturation) of *CBSYD1* is summarized in Table 2. The YD1 was purified to homogeneity by a two-step procedure (Fig. 3a, b), resulting in 40-fold purification and 12% activity recovery. Tricine SDS-PAGE analysis showed a single band of YD1 corresponding to a molecular weight of ~1 kDa (Fig. 3c). In the bioassay (in situ) step, YD1 presented a zone of inhibition corresponding to the same protein band as that observed in tricine SDS-PAGE. Stability studies revealed that YD1 remained completely stable at pH 4–9 and up to 80 °C, and its activity decreased sharply at or above 100 °C. The protease enzymes failed to alter the antimicrobial activity of the YD1 (Additional file 1: Table S1).

**Table 2:** Purification steps of YD1

Purification steps	Vol (mL)	Specific activity (AU/mL)	Total activity (AU)	Fold	Recovery (%)
Cell free supernatant	1000	800	8 × 10^5^	1	100
Ammonium sulphate	52	6400	3.328 × 10^5^	8	41.6
DEAE-Sepharose fast flow	7	25,600	1.792 × 10^5^	32	22.4
Sephadex G-25	3	32,000	0.96 × 10^5^	40	12

### Effects of YD1 in Synergism and *E. coli* cell

**Fig. 4:**
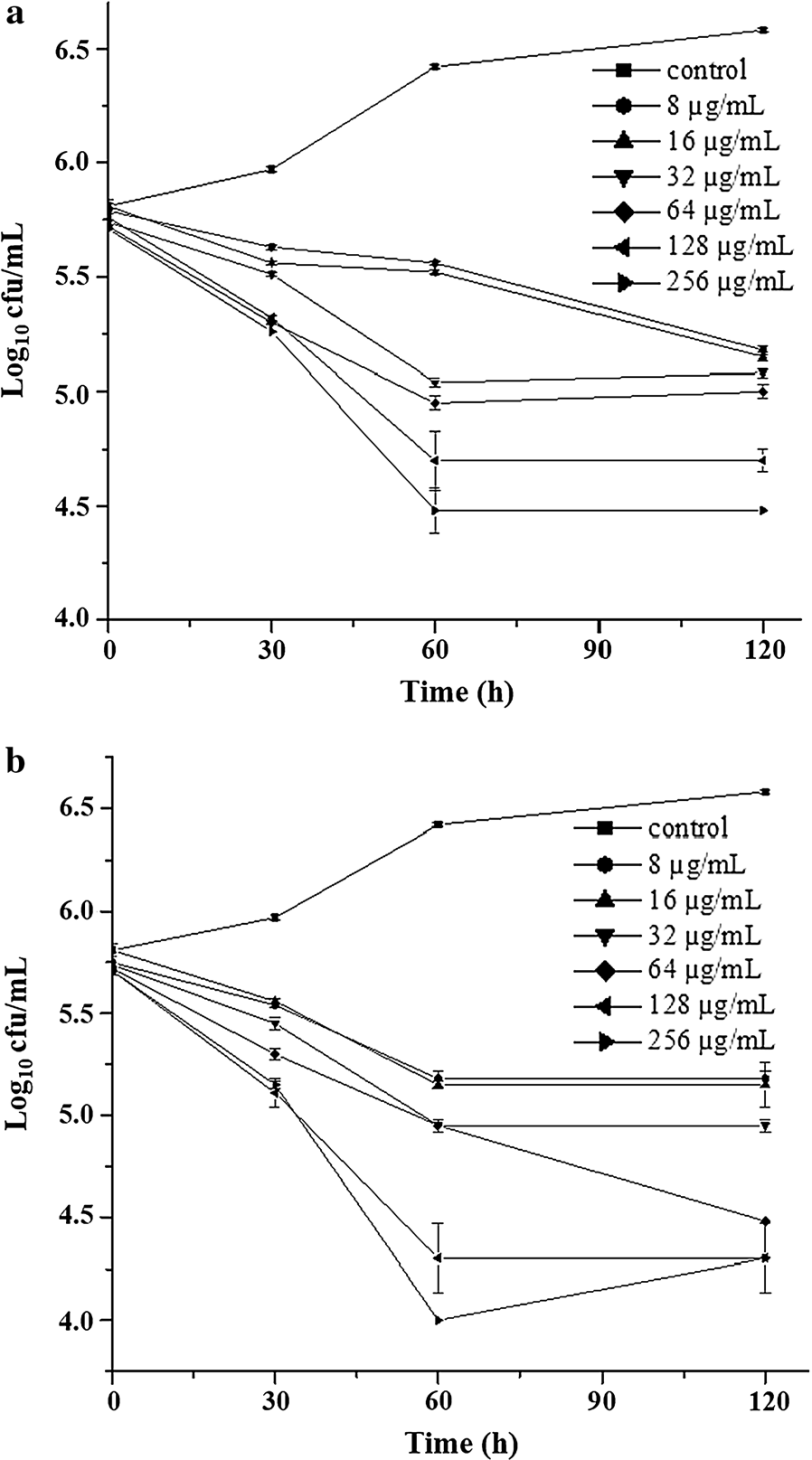
Effect of YD1 and erythromycin in combination on the rate of kill of *E. coli*. **a** Effect of YD1 (µg/mL) alone on *E. coli*.; **b** Effect of YD1 and erythromycin (µg/mL) in equal proportions on *E. coli*

**Fig. 5:**
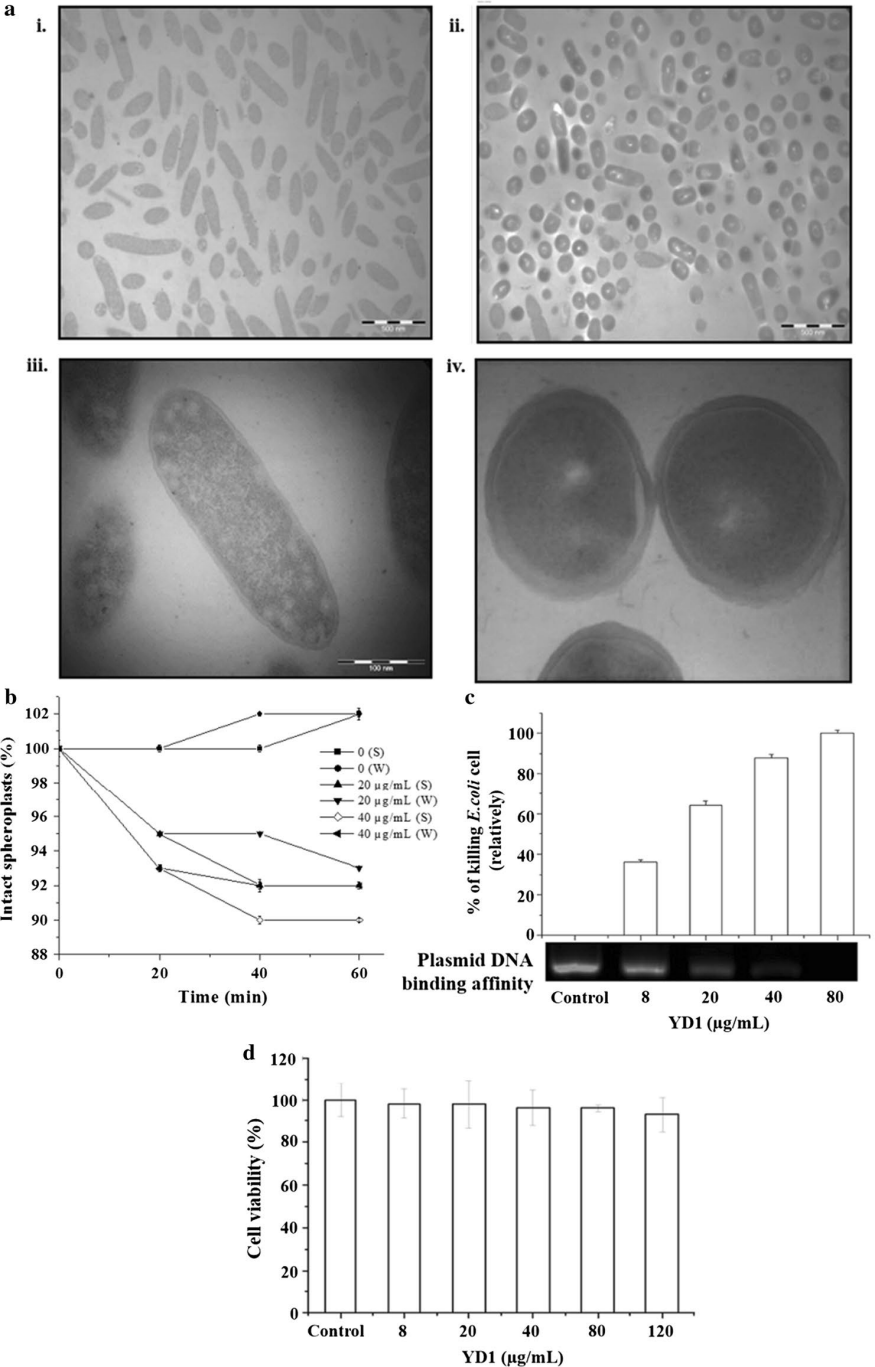
Transmission electron micrographs of *E. coli.* **a** (i) and (ii), untreated control of whole (×40 K zoom) and single cell (×100 K zoom) bacterial suspension, respectively, showing typical *E. coli* shapes; **a** (iii) and (iv), *E. coli’s* whole (×40 k zoom) and single cell (×100 K zoom) suspension, respectively, treated with 5× MIC (40 µg/mL) of YD1, showing *irregular shapes*. **b** Percentage of *E. coli* spheroplasts and whole cells remaining intact during exposure to YD1. Assays were performed in tris buffer with 20% w/v sucrose at OD 570 nm. **c** Plasmid DNA binding affinity measured for increasing amounts of YD1 (concentration ranging from 8–80 µg/mL). **d** Effects of YD1 on cell viability. Cell viability was measured after 24 h incubation. Survival rates were tested with MTT assay in Raw 264.7 cells. Raw 264.7 cells were incubated in the presence or absence of 8–120 µg/mL YD1 for 24 h. Each bar shows the mean ± S.D of three independent experiments performed in triplicate

Synergism effect was observed for YD1 and erythromycin with a mean FICI of 0.48, which suggested that YD1 may possess characteristics similar to erythromycin (Table 3; Fig. 4). Afterward, TEM was employed to investigate the effects of peptide treatment on membrane integrity and intracellular changes in *E. coli*. Typical cell membrane and intracellular contents were observed in the untreated bacterial cells [Fig. 5a (i), (ii)]. Cells of *E. coli* exposed to 5× MIC (40 µg/mL) of YD1 exhibited several changes after 4 h incubation. The treated cells showed a decrease in size and irregular shape; loss of cytoplasm and light staining; plasmolysis; and appearance of bubbles [Fig. 5a (iii)]. The microscopic studies have suggested that YD1 cause no disruption of the cytoplasmic membrane of *E. coli* cell. Further confirmation was established by observing the lack of lytic action of YD1 in whole cells of *E. coli* and >88% intact surviving spheroplasts [Fig. 5a (iv), b]. We hypothesized that the activity of YD1 was associated with the inhibition of macromolecular synthesis rather than with the damage to the bacterial cell wall. To clarify the molecular mechanism of action, the plasmid DNA binding affinity of YD1 was measured by analyzing the electrophoretic mobility of the DNA band. As shown in Fig. 5c, YD1 suppressed the migration of DNA in a dose-dependent manner, at 40 µg/mL concentration, the DNA migration was suppressed 80% and completely suppressed the migration of the DNA at 10× MIC (>80 µg/mL). Cytotoxicity studies showed that YD1 was not toxic to RAW 264.7 macrophage cells. As shown in Fig. 5d, YD1 did not exhibit cytotoxicity even at 120 μg/mL; ~90% of the cells were viable.

**Table 3:** Average FIC indices resulting from chequerboard titrations between YD1 and selected antibiotics

Antibiotic	Ceftriaxone Na		Erythromycin		Ciprofloxacin HCl	
Bacteria	Average FICI	Interpretation	Average FICI	Interpretation	Average FICI	Interpretation
*E. coli KCTC 1923*	2.19	Indifferent	0.48	Synergism	0.92	Indifferent
*MRSA B15*	1.15	Indifferent	0.51	Weak synergism/ indifferent	0.99	Indifferent

FIC index interpretations: ≤ 0.5, synergism; > 4, antagonism; > 0.5 to < 4.0 indifferent interaction

### Amino acid sequencing and computational analysis

**Fig. 6:**
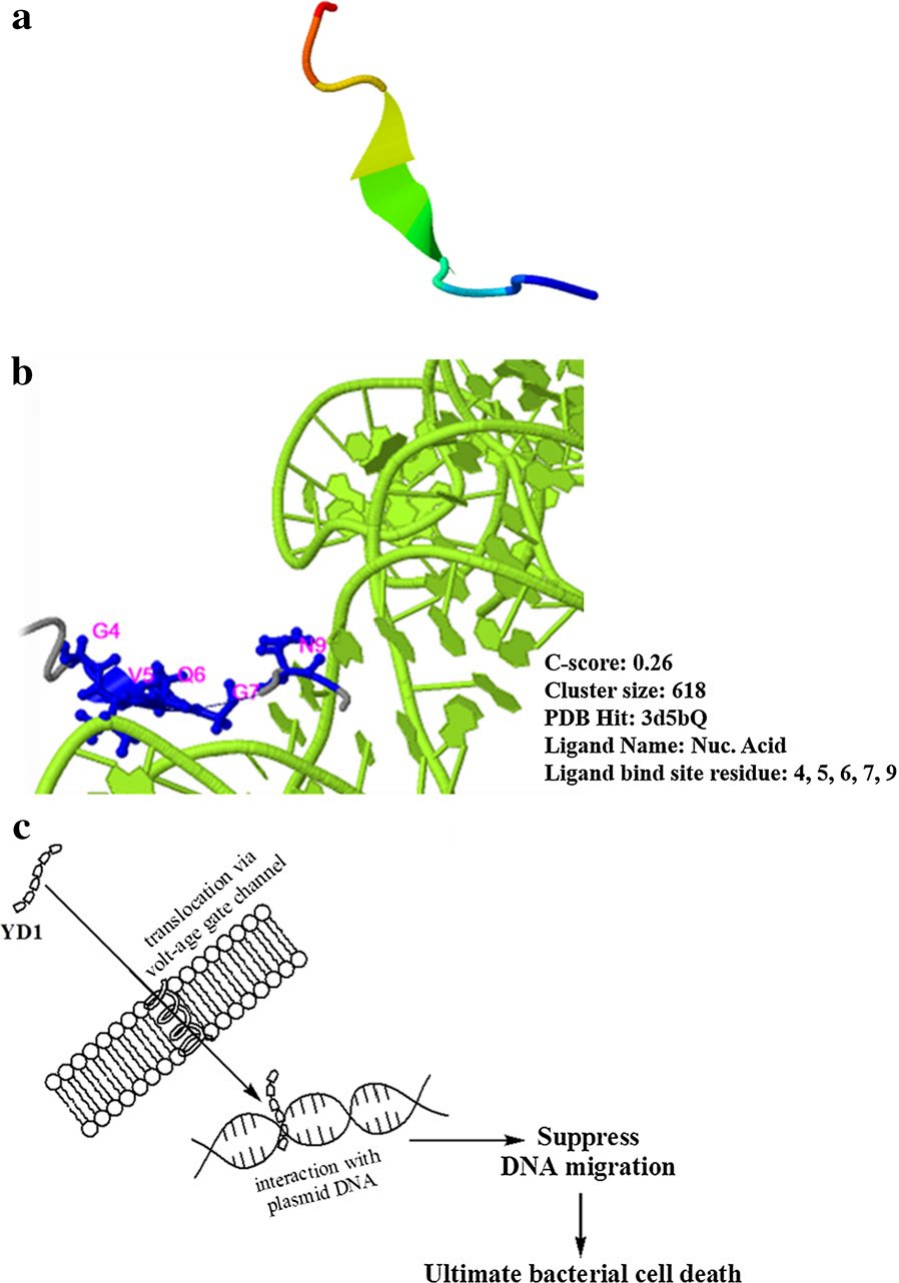
**a** Three-dimensional secondary structure projections of YD1. **b** The biological annotations of the target proteins by COACH based on the I-TASSER structure prediction. Predicted ligand binding sites of YD1. A diagram of hypothetical antimicrobial mechanism for novel AMP YD1 (**c**)

The amino acid sequence residues of YD1 were Ala-Pro-Lys-Gly-Val-Gln-Gly-Pro-Asn-Gly. The amino acid sequence was determined by Edman degradation method (Additional file 1: Figure S2) and analyzed using BLAST search against GenBank (http://www.ncbi.nlm.nih.gov/BLAST) and Antimicrobial Peptide Database (http://aps.unmc.edu/AP/main.php). After undergoing computational and sequence analysis using different servers suggested that YD1 possesses entirely novel amino acid sequence and a coil-shaped secondary structure (Fig. 6a) and has a net positive charge of +1 (Table 4). Comparison of YD1 sequence with other closely related AMP sequences, illustrated in Table 4, revealed differences. I-TASSER prediction analysis has suggested the presence of ligand binding sites of YD1 in a nucleic acid (NUC) (Fig. 6b).

**Table 4:** Alignment between antimicrobial peptides and YD1

Alignment		APD ID^a^	Similarity (%)	HR^b^ (%)	Net charge	Gram activity	MW^c^ (Da)	Source
		AP02344	40	14	+1	+, −	798.89	*Crocodylus siamensis*
		AP02672	36.84	31	+5	+, −	2114.6	*Hydrophylax bahuvistara*
		AP00320	35	52	+3	+, −	1941.3	*Uperoleia inundata*
		AP02606	33.33	50	+1	+, −	1106.3	*Rana* *limnocharis*
YD1	**-A-P-K-G-V-Q-G-P-N-G-**	Current study	100	20	+1	+, −	924.0	*B. amyloliquefaciens CBSYD1*

^a^Antimicrobial Peptide Database Identification

^b^Hydrophobic Residue

^c^Molecular weight (Dalton)

## Discussion

Screening and characterization of novel AMPs are attractive owing to their potential applications for therapeutic usage and in the food industry. In the present study, we identified, purified, and characterized an AMP from *strains* isolated from Korean fermented kimchi. Furthermore, we conducted a preliminary investigation of the antimicrobial mechanism of the purified AMP. Till date, a few AMPs producing strains have been isolated from kimchi (Mah et al. 2001; Teixeira et al. 2009; Wu et al. 2005). In our present study, we have reported the isolation of AMP producing bacterial strain, *CBSYD1*, from kimchi and studied the influence of several growth media and culture conditions on the production of AMP, and determined the optimized media (1% peptone, 2% maltose, and 0.01% CaCl_2_) that promotes greater production of YD1 over the commercially available media such as MRS and MH (Fig. 2). Growth media temperature and nutrients played a major role in the production of bacteriocin (Todorov et al. 2006). Bacteriocin activity was significantly higher at 37 °C, consistent with the results from Lisboa et al. on bacteriocin, produced by *B. amyloliquefaciens*, (Lisboa et al. 2010) and bacteriocin was very much stable at pH 4-9 and up to 80 °C, consistent with results by Todorov reports (Todorov et al. 2006). The protease enzymes failed to alter the antimicrobial activity of the YD1, which was not unusual (Korenblum et al. 2005). The homogeneity of YD1 was obtained by a two-step purification procedure, which resulted in 40-fold purification with 12% activity recovery. The purified YD1 has lower molecular weight compared to a bacteriocin-like substance (BLS) and some other *Bacillus* AMPs (Teixeira et al. 2009). YD1 was shown to be effective against both gram-positive and gram-negative bacteria as presented in Table 1. YD1 displayed a better antagonistic effect than reference antibiotics (bacitracin and vancomycin) against MDR pathogens such as *MRSA* and *VRE*. Antimicrobial effects of YD1 were prominent in comparison to other reports of AMPs against MDR, and non-MDR pathogens of *Bacillus* have been published (Dischinger et al. 2009; Sandiford and Upton 2012; Zheng and Slavik 1999).

The amino acid residues of YD1 were Ala-Pro-Lys-Gly-Val-Gln-Gly-Pro-Asn-Gly. APD search revealed that the closest (40%) similarity of YD1 was with an AMP Leucrocin I (AP02344) isolated from white blood cell extracts of crocodile, *Crocodylus siamensis* (Pata et al. 2011). Furthermore, sequence alignment suggested other AMPs (Table 4) showed ≤40% similarity. Sequences comparison with other AMP sequences revealed that YD1 possesses unique characteristics. Most of the AMPs are minuscule and strongly cationic (Yeung et al. 2011). Characteristics of the YD1 protein suggests that it is an AMP include: (1) small size (0.924 kDa) with 10 amino acids; (2) cationic character (net charge +1); and (3) theoretical pI of 8.80 (calculated according to amino acids sequences by ProtParam; http://web.expasy.org/protparam). Owing to its broad spectrum of antimicrobial activity and unique mechanism of action. Initial results from our study showed synergism between YD1 and erythromycin, a protein synthesis translocation inhibitor, indicating a relationship between their mechanisms of action (Odds 2003) and the property of YD1 to interact with intracellular macromolecules rather than the cell wall machinery. Using *E. coli* (10^6^ cfu/mL bacterial suspension) as a model organism, antimicrobial mechanism of action study was performed. TEM images showed morphological alteration in *E. coli* when treated with 5x MIC (40 µg/mL) of YD1 (Fig. 5a); while >88% of spheroplasts remained intact (Fig. 5b), 80% of *E. coli* cells died at the same YD1 concentration (Fig. 5c). This antibacterial action might result from effects on cellular metabolism. Kang et al. (2015) and Li et al. (2014) also reported the similar observation of morphological alteration of *E. coli* cells when treated with berberin and α-terpineol respectively (Kang et al. 2015; Li et al. 2014). In Fig. 5a, we demonstrated that YD1 possesses cell-penetrating and translocation ability, likely responsible for its antimicrobial activity without damaging bacterial cell wall or causing without cytotoxicity in RAW 264.7 macrophage cells (Fig. 5d). A recent review by Katrin and Neundorf (2011) enlisted the commonly used cell-penetrating peptides (CPP) and AMPs (Splith and Neundorf 2011). Furthermore, the positively charged residues in the α-helical sequence, and Arg-rich peptide, associate with the lipid phosphate groups to neutralize the Arg residue and allow the peptide translocation across the membrane (Amand et al. 2008; Boman 1995; Su et al. 2009). Recently, a report by Xie et al. (2011) suggested that the position of Pro residue in buforin II was more important than the overall α-helical content for the translocation (Xie et al. 2011). Consistent with these previous reports, YD1 AMP, a positively charged peptide, contains 3 glycines ([G^4,7,10^]) and 2 proline ([P^2,8^]) residues out of 10 amino acids. The antimicrobial potency of YD1 might not be determined solely by the coil-shaped secondary structure, but other factors such as the type of positive charge or the location of Pro might also be vital. In 2012, Jang and his co-workers (Jang et al. 2012) reported two cell-penetrating motifs Q-F-P–V-G and Q-W–P-V-G for peptides Buforin IIb and Buf IIIa, respectively. Analogously, Q-G-P-N-G is the likely expected cell-penetrating motif for YD1. Moreover, I-TASSER prediction analysis has suggested the presence of ligand binding sites in YD1 sequence for a nucleic acid (NUC) (Fig. 6b) which was consistent with the DNA binding affinity result shown in Fig. 5c.

In spite of the several proposed molecular processes regulated by AMPs, it is still unclear which, if any, of the hypothesized mechanisms, is responsible for their biological activity (Pálffy et al. 2009). Based on our findings, the antimicrobial mechanism of YD1 may be driven by cell-penetration and translocation inside the cell via a voltage-gated ion channel possibly (currently in process) and followed by interaction with bacterial plasmid DNA ultimately leading to bacterial cell death (Fig. 6c). After elucidation of complete structural information of the YD1 peptide (currently in progress), we will study the in-depth antimicrobial mechanism of action and synthesize AMP analogs.

In summary, the results presented here demonstrate the activity of the novel AMP YD1 purified from *Bacillus amyliquefaciens* subsp*. plantarum FZB42* isolated from Korean fermented kimchi. A broad-spectrum glycine-rich, low-molecular-weight AMP YD1 exhibits a unique antimicrobial mechanism of action characterized by its affinity for bacterial DNA, without damaging bacterial cell wall. Potent antimicrobial effects of YD1, in particular against resistant pathogens such as *MRSA, VRE*, and also *E. coli,* were observed. Our results collectively suggest that YD1 serve as a promising candidate for developing therapeutic agents for bacterial infections and endotoxin shock.

## Additional file

**Additional file 1: Table S1.** Effects of proteases on the stability of YD1; **Figure S1.** Effects of various nutrition sources in the production of antimicrobial compounds from *Bacillus CBSYD1*. (a) carbon sources (1%), (b) nitrogen sources (1%), (c) metal ion sources (0.01%), (d) 1% maltose and 0.01% CaCl_2_ were combined with variable amounts of peptone (0.5, 1, 1.5, and 2%). (e) 1% of peptone and 0.01% CaCl_2_ were combined with various percentage of maltose (0.5, 1, 1.5, and 2%). Culture was carried out in 250-mL flasks with 50 mL media, at pH 7 and 37 °C, with shaking at 160 rpm, **Figure S2.** The amino acid sequence of YD1 was determined by Edman degradation using a Procise Model 492 protein sequencer.

## Abbreviations

AMP: antimicrobial peptide
APD: anti-microbial peptide database
FICI: fractional inhibitory concentration index
KCTC: Korean collection for type culture
MDR: multidrug-resistant
MHA: Mueller hinton agar
MHB: Mueller hinton broth
MIC: minimal inhibitory concentrations
MRS: de Man-Rogosa-Sharpe
MRSA: *methicillin*-*resistant Staphylococcus aureus*
NUC: nucleic acid
SDS-PAGE: sodium dodecyl sulfate polyacrylamide gel electrophoresis
TEM: transmission electron microscopy
VRE: *vancomycin*-*resistant enterococci*
WDCM: World Data Centre for Microorganisms
