# Supplementary material for: Glycin-rich antimicrobial peptide YD1 from B. amyloliquefaciens, induced morphological alteration in and showed affinity for plasmid DNA of E. coli
Source: AMB Express. 2017 Jan 3;7:8. doi: 10.1186/s13568-016-0315-8 (PMC5209312; doi:10.1186/s13568-016-0315-8)
Supplement: Supplementary file 1 — Additional file 1: Table S1. Effects of proteases on the stability of YD1; Figure S1. Effects of various nutrition sources in the production of antimicrobial compounds from Bacillus CBSYD1. (a) carbon sources (1%), (b) nitrogen sources (1%), (c) metal ion sources (0.01%), (d) 1% maltose and 0.01% CaCl2 were combined with variable amounts of peptone (0.5, 1, 1.5, and 2%). (e) 1% of peptone and 0.01% CaCl2 were combined with various percentage of maltose (0.5, 1, 1.5, and 2%). Culture was carried out in 250-mL flasks with 50 mL media, at pH 7 and 37 °C, with shaking at 160 rpm, Figure S2. The amino acid sequence of YD1 was determined by Edman degradation using a Procise Model 492 protein sequencer. [file 13568_2016_315_MOESM1_ESM.pdf]

ADDITIONAL SUPPORTING FILES

Journal Name: AMB Express

Glycin-rich antimicrobial peptide YD1 from *B. amyloliquefaciens*, induced morphological alteration in and showed affinity for plasmid DNA of *E. coli*

Md. Saifur Rahman [a], Yun Hee Choi [a], Yoon Seok Choi, and Jin Cheol Yoo*

Department of Pharmacy, College of Pharmacy, Chosun University, Gwangju 501-759, Republic of Korea

Author's e-mail address: saifpharmacist@outlook.com (M.S.Rahman), unichy@nate.com (Y.H.Choi), ztakeiteasy@gmail.com (Y.S.Choi), and jcyu@chosun.ac.kr (J.CH. Yoo*)

[a] Both authors contributed equally to this work.

Running title: Novel antimicrobial peptide YD1 with a unique mechanism of action

[a]Department of Pharmacy, College of Pharmacy, Chosun University, Gwangju 501-759, Korea

*Correspondence: Jin Cheol Yoo, Department of Pharmacy, College of Pharmacy, Chosun University, Gwangju 501-759, Korea

Tel.: +82 62 230 6380; Fax: + 82-62-227-3963; E-mail: jcyu@chosun.ac.kr

ADDITIONAL SUPPORTING FILES

Table S1: Effects of proteases on the stability of YD1.

**Proteases stability**		
Digestive enzyme	Concentration	Residual activity
Lipase	1mg/ml	100
	2mg/ml	100
Trypsin	1mg/ml	100
	2mg/ml	100
Pronase	1mg/ml	100
	2mg/ml	100

Figure S1:

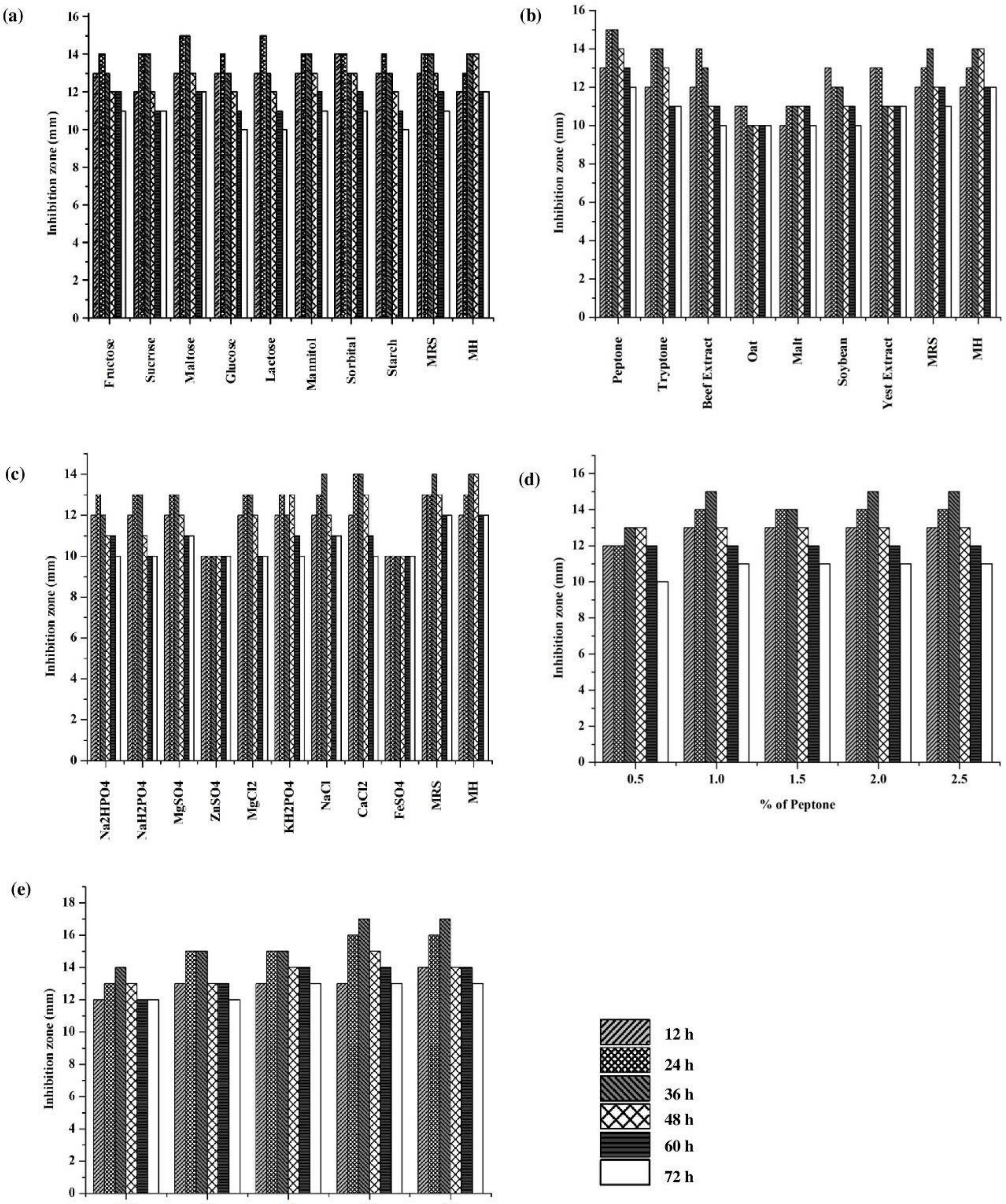

Figure S1: Effects of various nutrition sources in the production of antimicrobial compounds from *Bacillus CBSYD1*. (a) carbon sources (1 %), (b) nitrogen sources (1 %), (c) metal ion sources (0.01 %), (d) 1 % maltose and 0.01 % $CaCl_2$ were combined with variable amounts of peptone (0.5, 1, 1.5, and 2 %). (e) 1% of peptone and 0.01% $CaCl_2$ were combined with various percentage of maltose (0.5, 1, 1.5, and 2 %). Culture was carried out in 250-mL flasks with 50 mL media, at pH 7 and 37 °C, with shaking at 160 rpm

Figure S2

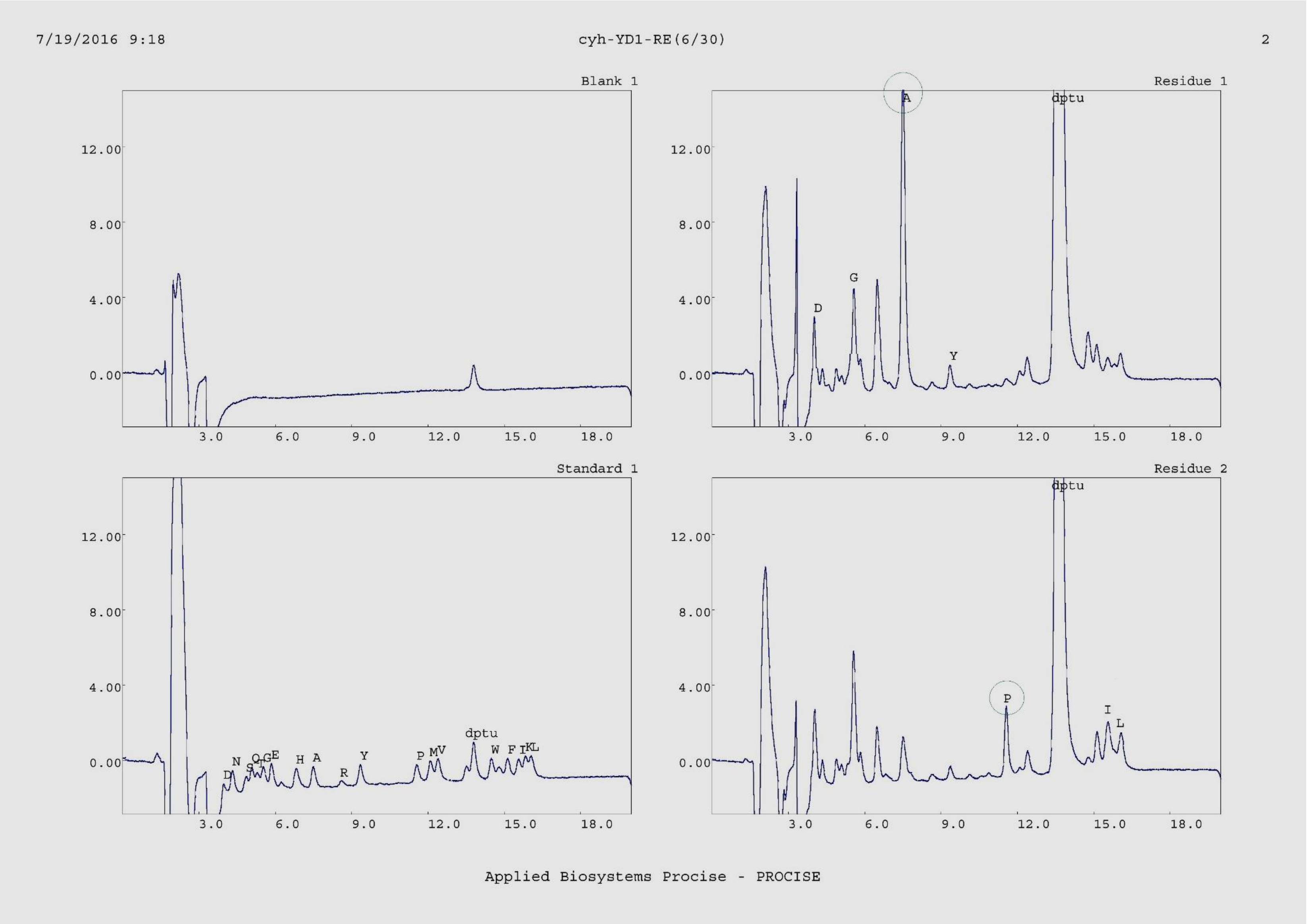
7/19/2016 9:18
cyh-YD1-RE(6/30)
2
Blank 1
Residue 1
Standard 1
Residue 2
12.00
8.00
4.00
0.00
3.0
6.0
9.0
12.0
15.0
18.0
D
G
A
Y
dptu
N
S
Q
T
G
E
H
R
P
M
V
W
F
I
K
L
Applied Biosystems Procise - PROCISE

ADDITIONAL SUPPORTING FILES

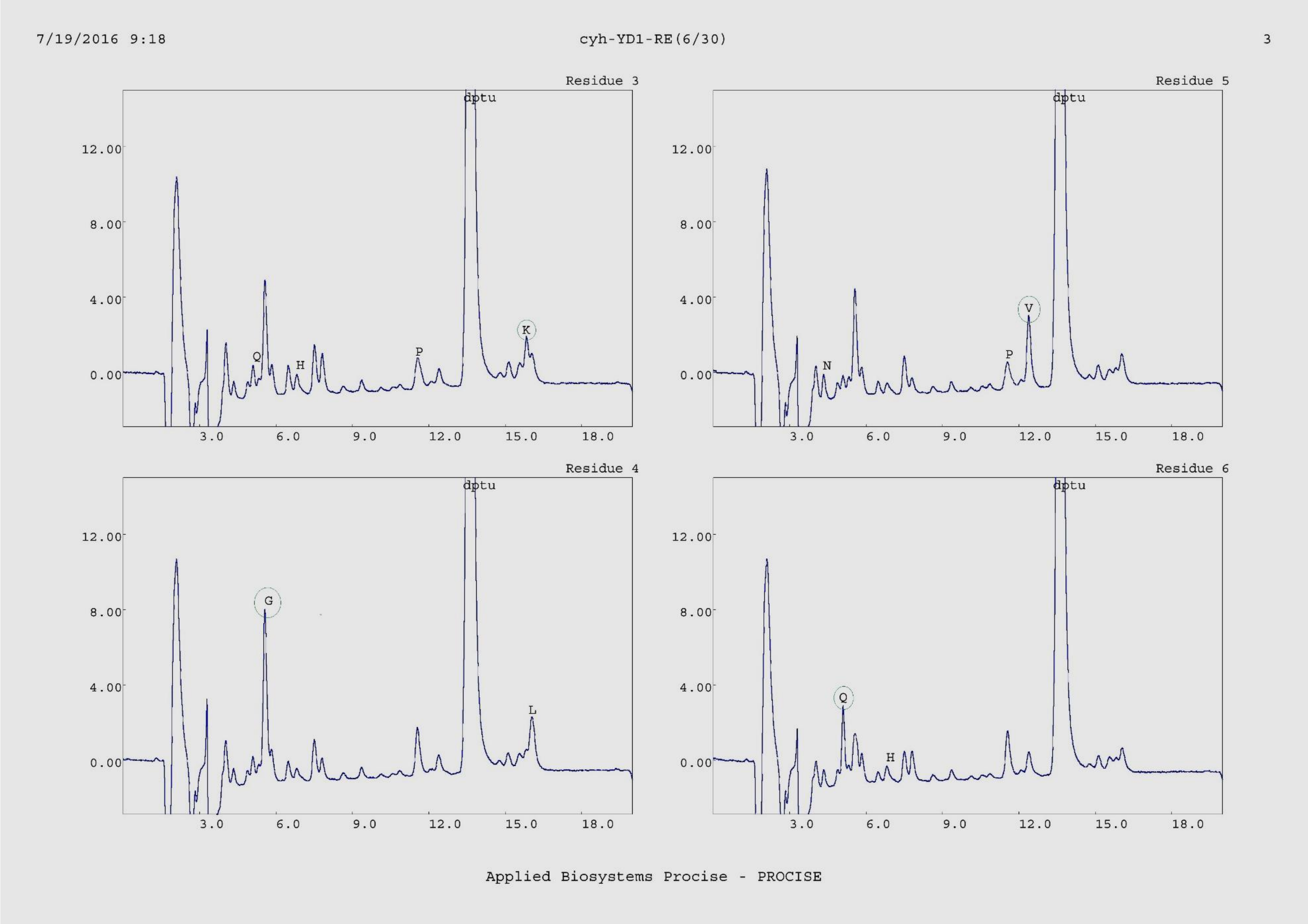

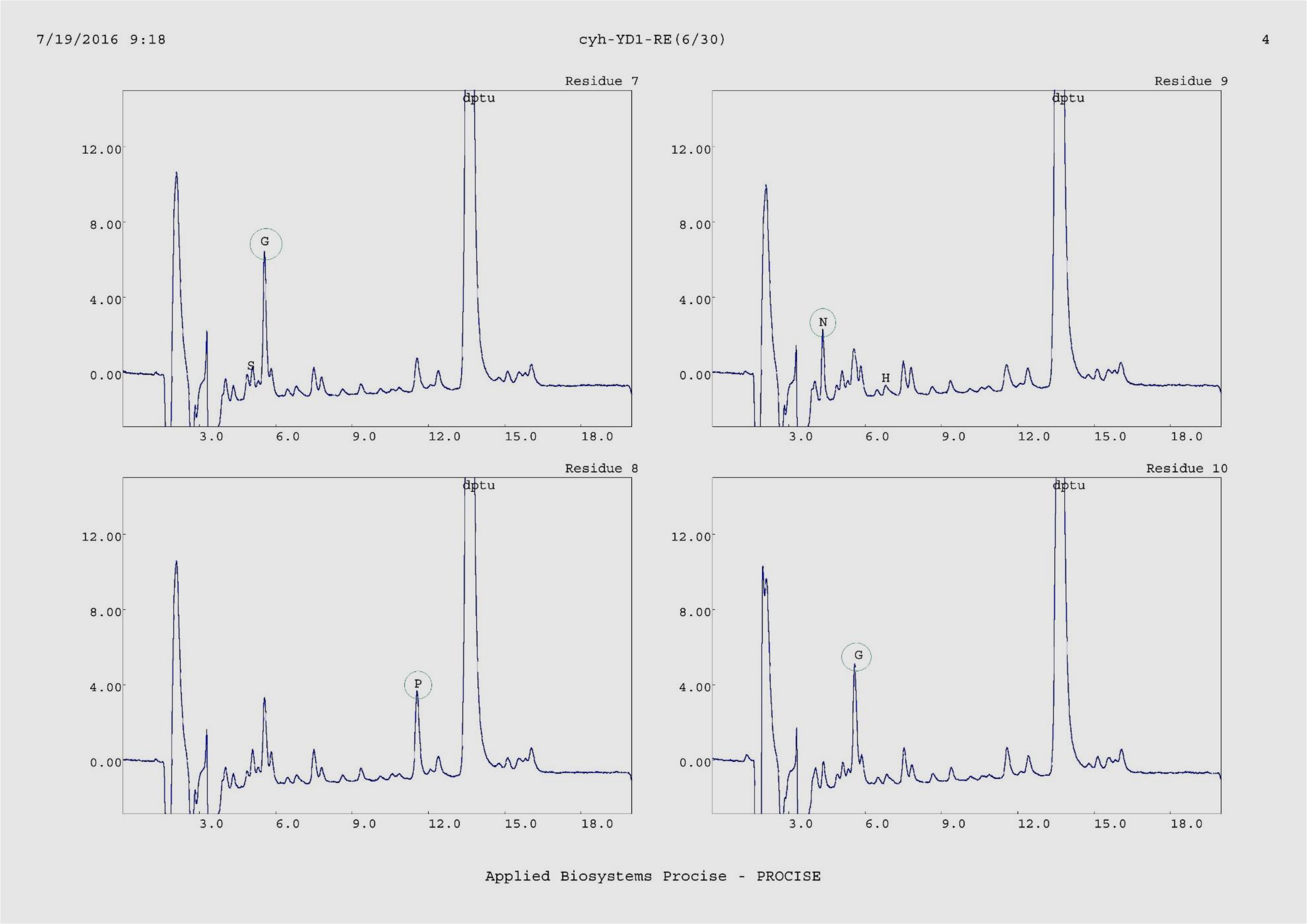

Figure S2: The amino acid sequence of YD1 was determined by Edman degradation using a Procise Model 492 protein sequencer.
